# Round the Hospitals

**Published:** 1917-01-20

**Authors:** 


### ROUND THE HOSPITALS.

The practice of "make-believe" once begun seems difficult to shed. Is there one of our readers, we wonder, who has ever heard of the Trained Nurses' Protection Committee, which, we understand, is the latest **authority** on which the Lady rests her claim to represent the nursing profession as a whole, which is "clamouring "against its oppressors for the full measure "of professional responsibility which is its "right?" We have not been able to discover from any cuttings agency, nor have we found in any publication, "an expression of the indignation "of British nurses concerning indignities heaped "upon them by the College Council," which that Council is now supposed to have removed in its agreement with the Royal British Nurses' Association. The Trained Nurses' Protection Committee has successfully hidden its light under a bushel, for no one, so far as we have been able to discover, is aware of any wide publicity which it has given to the indignation of British nurses. Might we inquire what relation this latest "**authority**" bears to the Trained Nurses' Economic League, to the Nurses' Defence Union, and to the other "make-believe" authorities which appear to have slumbered of late, if they have not found a place in the limbo of forgotten things? How many of the members of these latter "authorities" have become members of the infant Trained Nurses' Protection Committee, the latest progeny of make-believe?

The *Weekly Irish Times* has done good service to the nursing profession in Ireland by devoting much space to a discussion of the College of Nursing and the views of the opponents of the College in Ireland. There will be meetings of the College of Nursing in Belfast on the 29th inst., and in Dublin on the 27th inst. That at the Royal College of Physicians, Dublin, the president, Dr. O'Carroll, in the chair, which we announced last week, will be held at 5 p.m. Miss Cox-Davies and Miss Rundle should have a large and attentive audience, for the discussion promises to be interesting and instructive. From a statement in the *Weekly Irish Times* of the 13th instant it appears, from the views expressed by the chairman, Dr. W. I. de Courcy Wheeler, of the City of Dublin Nursing Institution, who is also visiting surgeon to Mercers' Hospital, Dublin, that he inclines to think that the circumstances of the training and qualification, and of the conditions under which nurse-training is given in Ireland, differ so materially from those in England as to make it desirable that, great caution should be exercised by Irish nurses before they come to a decision in the matter. Dr. Wheeler suggests that if Ireland had a Royal Charter for nurses and, if it is desired, an Irish College of Nursing established by Act of Parliament, seeing that Irish M.P.s have always voted solid in favour of registration, Ireland might lead and not follow, and he sees no reason why Ireland, properly organised, should lag behind.

In any case Dr. Wheeler is strongly of opinion that it is eminently desirable to organise nurses in Ireland, seeing that the fees paid for training are in many cases far too high, and that where the training is free, or nearly so, it is not consequently in any sense of an inferior type. The hours and rate of remuneration of nurses in Ireland demand the revision, which organisation would secure. No doubt those who know most about Irish questions, and the difficulties which sometimes accompany them, may incline to the feeling that the acceptance of Dr. Wheeler's views has something to be said in its favour, and that if they prevail that is no reason why an Irish organisation should not ultimately become affiliated with the College of Nursing. We hope all these points may be threshed out at the meeting, and that whatever decision is come to it will be one which will best accord with Irish feeling, and tend directly to secure the maximum benefit for Irish nurses. On the other hand, the simplest and speediest way for Ireland to bring about the registration of nurses and its statutory recognition will undoubtedly be, for Irish nurses to agree to follow the example of Scotland by appointing representatives on the Council of the College of Nursing. They could then arrange, under the College scheme, to have an Irish Board, and to carry out Irish registration, as is being done at the present time with success in Scotland. We wish Miss Cox-Davies and Miss Rundle every success, and hope the personal service they are rendering to Irish nurses and the nursing profession may be recognised by both. A College of Nursing Committee has been appointed in Ireland, consisting of Miss Eddison, matron Royal City of Dublin Hospital; Miss Egan, president Irish Matrons' Association; Miss Hill, matron Adelaide Hospital; Miss McGivney, matron Mater Misericordiæ Hospital; Miss O'Brien, 67 Lower Leeson Street; Miss Shuter, Ivanhoe Nursing Home; and Miss Reed, Ivanhoe Nursing Home.

An important meeting in support of the College of Nursing will be held in Leeds at the Philosophical League Hall on Friday, 19th instant, at 3 p.m., when the Hon. Arthur Stanley, M.P., and Miss Swift, R.R.C., will attend and address the meeting.

